# Validity and reproducibility of a short food frequency questionnaire among patients with chronic kidney disease

**DOI:** 10.1186/s12882-017-0695-2

**Published:** 2017-09-15

**Authors:** Aurélie Affret, Sandra Wagner, Douae El Fatouhi, Courtney Dow, Emmanuelle Correia, Maryvonne Niravong, Françoise Clavel-Chapelon, Julie De Chefdebien, Denis Fouque, Bénédicte Stengel, on behalf of CKD-REIN study investigators, Bénédicte Stengel, Christian Jacquelinet, Bruce Robinson, Ziad A. Massy, Christian Combe, Denis Fouque, Maurice Laville, Luc Frimat, Carole Ayav, Serge Briançon, Christophe Pascal, Yves-Edouard Herpe, François Deleuze, Joost Schanstra, Pascal Morel, Marie-Christine Boutron-Ruault, Guy Fagherazzi

**Affiliations:** 1 Inserm U1018, Center for Research in Epidemiology and Population Health (CESP), Villejuif, France; 2 Univ Paris-Sud, Villejuif, France; 3 UVSQ, Villejuif, France; 4 FCRIN INI-CRCT, Lyon, France; 5 Centre Hospitalier Lyon Sud, Lyon, F-69495 Pierre-Bénite, France; 6 CarMeN, Inserm UMRS 1060, Inserm, Univ Lyon-Sud, Lyon, 69921 Oullins, France; 7 Center for Research in Epidemiology and Population Health (CESP), INSERM (Institut National de la Santé et de la Recherche Médicale) U1018, Team 9, Health across generations, Gustave Roussy Institute, 114 rue Edouard Vaillant, 94805 Villejuif Cedex, France

**Keywords:** Chronic kidney disease, Short food frequency questionnaire, Validity, Reproducibility, Diet, Dietary assessment

## Abstract

**Background:**

A balanced diet is essential to slowing the progression of chronic kidney disease (CKD) and managing the symptoms. Currently, no tool is available to easily and quickly assess energy and macronutrient intake in patients with non end-stage CKD.

We aimed to develop and evaluate the validity and reproducibility of a new short 49-item food frequency questionnaire (SFFQ) adapted to patients with CKD.

**Methods:**

The CKD-REIN study is a prospective cohort that enrolled 3033 patients with moderate or advanced CKD from a national sample of nephrology clinics. A sub-sample of 201 patients completed the SFFQ twice, at a one-year interval and were included in the reproducibility study. During this interval, 127 patients also completed six 24-h recalls and were included in the validity study. Main nutrient and dietary intakes were computed. Validity was evaluated by calculating crude, energy-adjusted and de-attenuated correlation coefficients (CC) between FFQ and the mean of the 24-h recall results. Bland-Altman plots were performed and cross-classification into quintiles of consumption of each nutrient and food group was computed. Reproducibility between the two SFFQs was evaluated by intraclass CC (ICC).

**Results:**

Regarding validity, CC ranged from 0.05 to 0.79 (unadjusted CC, median: 0.40) and 0.10 to 0.59 (de-attenuated CC, median: 0.35) for food group and nutrient intakes, respectively. Five of the most important nutrients of interest in CKD, i.e. protein, calcium, phosphorus, potassium, and sodium had de-attenuated CC of 0.46, 0.43, 0.39, 0.32, and 0.12, respectively. The median of classification into the same or adjacent quintiles was 68% and 65% for food and nutrient intakes, respectively, and ranged from 63% to 69% for the five nutrients mentioned before. Bland-Altman plots showed good agreement across the range of intakes. ICC ranged from 0.18 to 0.66 (median: 0.46).

**Conclusions:**

The CKD-REIN SFFQ showed acceptable validity and reproducibility in a sample of patients with CKD, notably for CKD nutrients of importance. It can now be used in large-scale epidemiological studies to easily assess the relations between diet and CKD outcomes as well as in clinical routine. It may also serve as a basis for the development of FFQs in international CKD cohort networks.

**Electronic supplementary material:**

The online version of this article (10.1186/s12882-017-0695-2) contains supplementary material, which is available to authorized users.

## Background

Chronic Kidney Disease (CKD), defined by the presence of abnormalities in kidney structure or function for a period greater than 3 months, is common [1]. It is associated with high risks of mortality and progression to end-stage renal disease (ESRD), for which kidney replacement therapy (dialysis) or transplantation is required. To avoid progression to ESRD, prevention of CKD progression and management of the symptoms through nutrition are essential [2]. Indeed, epidemiological and clinical evidence have shown links between several micronutrients and CKD [3]. In this context, it is currently recommended (but rarely achieved) to reduce dietary protein intake (DPI) to 0.8 g/kg/day from CKD stage 3 [1, 4], even if the DPI effect on the progression of CKD is still debated [5–7]. Calcium, phosphorus, potassium and sodium chloride intakes also need to be monitored, from CKD stage 3, due to their relation with mineral, bone and cardiovascular CKD complications [1, 4]. Finally, because more than 40% of CKD patients have diabetes, they are encouraged to limit the specific nutrients diabetic patients are advised to limit (i.e. simple sugars and saturated fats) [8]. In terms of dietary patterns, the Dietary Approaches to Stop Hypertension (DASH) diet and the Mediterranean diet, both low in dietary acid load, have been associated with favorable CKD outcomes [3].

Despite these numerous and complex dietary recommendations and findings, very few CKD patients with non-ESRD have a dietetic follow-up, and little is known about their food consumption.

It is therefore essential to examine the relationship between dietary factors and outcomes in patients with CKD [9]. Food Frequency Questionnaires (FFQs) have been previously developed in the CKD context but most of them are long questionnaires (around 150 items) [1, 10–13] or focus on the estimation of specific nutrient/food intakes [14, 15]. One SFFQ of 76 food items was developed in the Brazilian context [16].

A consortium of 6 cohorts (E3N [17], E4N [18], CKD-REIN [13], i-Share [19], Elfe [20] and Psy-COH) was created to obtain a unique Short Food Frequency Questionnaire (SFFQ), useful to quickly assess the diet in several population subgroups: patients with CKD (CKD-REIN cohort), adolescents, students, adults, the elderly and patients with mental disorders.

Existing SFFQs have not been suitable to quickly assess the diet in several French population subgroups: i) the SFFQ developed by Vercambre et al. [21] was developed for senior women and focuses on nutrients of interest for this population, ii) one third of the items of the SFFQ developed by Giovannelli et al. [22] are not valid and no food composition table is available to study nutritional intakes, iii) the SFFQ developed by Barrat et al. [23] refers to past week intakes, does not take seasonal variability into account and does not focus on nutrients of interest in a CKD context. Therefore, the consortium decided to develop a new and unique SFFQ (40 items) adapted to several population subgroups of interest. They also agreed that few additional specific questions (10 items max., 9 in the present study) could be added to the questionnaire to aid in the estimation of some nutrients of interest, in the context of specific populations such as CKD patients.

The CKD-REIN (CKD-Renal Epidemiology and Information Network) cohort was chosen to be the first pilot study for evaluating the SFFQ feasibility and validity. Before using a newly developed or modified FFQ, it must first be validated to be considered as an acceptable method of dietary assessment [24]. The aim of the present study was to study the reproducibility of the newly developed SFFQ, and evaluate its validity against six 24-h recalls, in a sample of patients with moderate or advanced CKD.

## Methods

### Study population and design

The CKD-REIN study is a clinical-based prospective cohort that included 3033 adult patients with moderate or advanced CKD from a nationally representative sample of nephrology facilities between July 2013 and March 2016. The design and methods for this cohort have been described in detail elsewhere [25]. Between April and June 2014, participants who were included in the CKD-REIN study were informed about the design of the present reproducibility and validity study and were invited to participate. In total, 301 participants were volunteers, gave informed consent and were mailed the newly developed SFFQ. They were asked to complete the SFFQ twice, at a 1 year interval, in June 2014 and 2015. 244 participants completed the SFFQ once and 208 participants completed it twice. Participants who under- or over- reported energy intake in one of the SFFQs were excluded as previously described [26]: they were in the top and bottom 1% of the energy intake to energy requirement ratio distribution. Energy requirement was calculated as follows: Basal Metabolic Rate (BMR)* Physical Activity Level (the cutoff value of 1.55 for a minimal activity level was chosen [27]). BMR was computed on the basis of sex, age, height and weight, using the Schofield formula [28]. A total of 201 participants were included in the reproducibility study. Among them, 127 patients agreed to answer six 24-h recalls and were therefore included in the validity study (a flow diagram is presented in Additional file 1).

### Short food frequency questionnaire

The food list of the SFFQ was defined on the basis of existing national food questionnaires [21–23, 29–32] and data from the second national study of individual food intakes of French adults (INCA 2) [33]. The SFFQ was self-administered, completed at home and then returned by mail. The questionnaire asked participants to report their usual dietary intake over the past year and was divided into two parts. In total, 49 items were used to obtain the nutritional data (see Additional file 2).

The first part was composed of 40 food group items. It quantified consumption by frequency (never or less than once a month, *x* times a day, *x* times a week or *x* times a month) and portion sizes per food group item. Photos previously validated [34] were directly integrated into the questionnaire to help participants estimate the consumed quantities of 21 food items (see Additional file 3). Most of the time, there were three photos showing increasing portion sizes with five possible answers (less than the lowest portion, the lowest portion, an intermediate portion, the biggest portion, more than the biggest portion). For items with no photo, participants were asked to quantify their consumption with respect to a standard portion size (typical household measurements such as spoons or standard units such as individual pots of yogurts).

The second part was specific to the study population of patients with CKD. It was composed of nine questions, with the objective of estimating the intake of some nutrients of specific interest as best as possible in this population, such as protein, calcium, phosphorus, potassium, and sodium. Seven of the nine questions provided more detailed information on some food group items asked in the first part of the questionnaire. The two remaining questions enquired about extra- salt added when cooking and the consumption of processed foods.

All the information collected was used to calculate daily intakes of each food group. Frequencies were converted into numbers of servings per day and multiplied by the declared portion size. An ad hoc composition table was developed using data from the INCA2 French representative population survey [33] to estimate the percentage of contribution of each food included in a food group item. Nutritional data were then obtained using the French food composition database established by the French Data Centre on Food Quality (Ciqual, last updated in 2013) [35].

Besides nutritional information, the SFFQ elicited information on sex, birth date, and anthropometric data. It also questioned the participants about dietitian visits and eventual changes of food habits during the past year due to particular situations (diet, pregnancy, move, surgery, depression,…).

### 24-h recalls

The reference method used to compare results from the SFFQ consisted of six 24-h recalls carried out each 2 months, during the year between the first and the second administration of the SFFQ. Study participants were asked to recall all foods and beverages consumed on the previous day (due to logistics, data for Saturdays were collected on Mondays). Participants were not informed in advance of the day of the recall. All weekdays and all seasons were covered by the recalls in order to account for intra-individual variation. Phone interviews were carried out by a trained dietitian who entered the food data into the Nutrilog Software (v2.70d). These data were instantaneously converted into nutrient intakes by the software, using the Ciqual food composition database [35]. A validated photo album of 42 foods [32] was previously sent to the participants’ homes in order to help them quantify the amount of consumed food during the phone interview.

### Statistical analysis

We computed descriptive statistics (means and standard deviations) for nutrients and foods for both SFFQs and the average of the six 24-h recalls. Wilcoxon signed rank tests were performed to study differences between mean values.

### Validity

To study relative validity, data evaluated by the second SFFQ were compared with the mean of the six 24-h recalls, since both methods covered the same period of time. A list of concordance was established between food group items from the SFFQ and food items provided by 24-h recalls.

Regarding food groups, unadjusted Spearman’s correlation coefficients were calculated. Regarding nutrient intakes, unadjusted and energy-adjusted Pearson correlation coefficients were calculated. Energy-adjusted coefficients, corrected for attenuation due to within-person variation in the reference method (de-attenuated coefficients) [36, 37], were also produced. Energy adjustment was performed using the residual method [37]. To improve the normal distribution, nutrient intakes were logarithmically transformed before analysis.

We examined the level of agreement in ranking subjects between the two methods through cross-classification into quintiles, in terms of food group and nutrient intakes. Misclassification was defined as the percentage of participants classified in the lowest quintile in the SFFQ and the highest quintile in the 24-h recalls and vice versa. Due to several food groups with a proportion of non-consumers > 20%, cross-classification into three classes was performed. Classes were defined as follows: class = 1 for null consumption; class = 2 for consumption below or equal to the median value in consumers, class = 3 for consumption over the median value in consumers. For food groups with a proportion of non-consumers < 20%, cross-classification into tertiles was also performed.

We also evaluated agreement between the SFFQ and the six 24-h recalls performing Bland-Altman plots on energy-adjusted values [38–40]. Mean differences between the two assessment methods were plotted against the average estimation of the two methods. The 95% limit of agreement was calculated as the mean difference ± 1.96 SD.

### Reproducibility

To evaluate reproducibility, data obtained from the first and second SFFQ were compared. Regarding food groups, unadjusted Spearman’s correlation coefficients and intraclass correlation coefficients (ICC) were estimated. Regarding nutrient intakes, unadjusted and energy-adjusted Pearson correlation coefficients as well as ICC were calculated. Nutrient intakes were logarithmically transformed before analysis, to improve the normal distribution.

The level of agreement in ranking subjects between the two SFFQs in terms of food group and nutrient intakes was examined through cross-classification into quintiles.

All statistical analyses were performed on SAS 9.4 (SAS Institute Inc., Cary, NC, USA). A *P-*value < 0.05 was considered statistically significant.

## Results

Baseline characteristics of the participants included in the validity and reproducibility studies were similar **(**Table 1 **)**. Briefly, 35.3% of the participants were women. The mean age was 65.3 ± 11.8 years and the mean BMI was 28.1 ± 5.5 kg/m^2^. The participants had moderate to advanced CKD: 56.2% stage 3 and 43.8% stage 4. Baseline mean Glomerular Filtration Rate (GFR) was 31.6 ± 11.8 ml/min/1.73m^2^. 39.3% of participants had diabetes (mean HbA1c = 6.3 ± 1.1% and mean glycaemia = 6.1 ± 1.7 mmol/l). Participants lived in all regions of France, with significant proportions in the Eastern North and Western South.

**Table 1:** Descriptive characteristics of the subjects included in the validity and reproducibility study

	Validity	Reproducibility
	*N* = 127	*N* = 201
	Mean (SD)	
Sex (% women)	37.8	35.3
Age (years)	67.4 (10.1)	65.3 (11.8)
BMI (kg/m^2^)	28.0 (5.6)	28.1 (5.5)
CKD stage	%	
Stage 3	58.3	56.2
Stage 4	41.7	43.8
GFR (ml/min/1.73m^2^)^a^	Mean (SD)	
	30.9 (10.7)	31.6 (11.8)
	Mean (SD)	
With diabetes (%)	37.8	39.3
HbA1C (%)^a^	6.3 (1.1)	6.3 (1.1)
Glycaemia (mmol/l)^a^	6.0 (1.7)	6.1 (1.7)
Area of residence	%	
Western North	3.9	6.5
Eastern North	37.8	31.3
Western South	38.6	35.3
Eastern South	10.2	12.9
Paris and suburbs	9.4	13.9
Distribution of 24-h recall days	Mean (SD)	
Weekday	4.3 (0.5)	
Weekend	1.7 (0.5)	
Autumn/winter	2.8 (0.6)	
Spring/summer	3.2 (0.6)	

*BMI* Body Mass Index, *CKD* chronic kidney disease, *GFR* Glomerular Filtration Rate

^a^GFR has been measured at patients’ inclusion in the CKD-REIN study; HbA1C and Glycaemia have been measured at a point close to SFFQ1 fill in (average of 2.7 and 2.6 months before SFFQ1 administration in the validity and reproducibility studies, respectively)

### Validity

#### Food groups

Dietary intakes estimated by the SFFQ2 and the mean of the six 24-h recalls were mostly comparable **(**Table 2 **)**. However, the SFFQ overestimated some food groups: ‘whole-grain pasta, rice and wheat’, ‘legumes’, ‘milk’, ‘fruit juice’, ‘sweet beverages’, and ‘artificially-sweetened beverages’. The SFFQ also underestimated the following food group: ‘other alcoholic beverages’. These food groups had a large number of non-consumers (>20% of participants according to SFFQ2).

**Table 2:** Relative validity of the SFFQ for food groups (*n* = 127)

	Daily intakes			Spearman correlation coefficients	Cross-classification of food group distributions		
	24-hour recalls	SFFQ 2	SFFQ2-24-hour recalls	SFFQ2 *vs.* means of six 24-hour recalls			
	Mean in grams (foods) or ml (beverages) (SD)		Means of differences, in grams (foods) or ml (beverages) (SD)	Unadjusted	% of subjects classified in same tertile^a^	% of subjects classified in same or adjacent quintile	% of misclassified subjects
Whole-grain bread and substitutes^b^	20.3 (40.3)	29.9 (53.3)	9.6 (64.3)	**0.15**	48	.	.
White bread and substitutes	83.3 (63.4)	93.2 (91.7)	9.9 (75.1)	0.49	51	73	2
Breakfast cereals^b^	0.9 (6.0)	1.5 (6.1)	0.6 (5.6)	0.65	95	.	.
Whole-grain pasta, rice and wheat^b^	2.8 (18.4)	20.0* (35.1)	17.1 (32.4)	0.19	50	.	.
White pasta, rice and wheat	67.8 (58.9)	43.8* (45.9)	-24.0 (56.1)	0.39	47	66	2
Legumes^b^	7.0 (16.7)	26.9* (44.0)	19.8 (47.7)	**0.05**	24	.	.
French fries and other fried tubers^b^	8.3 (12.2)	11.1 (15.7)	2.8 (18.7)	**0.17**	34	.	.
Potatoes and other tubers (not fried)	61.0 (51.9)	52.5 (49.9)	-8.4 (62.6)	0.22	42	66	6
Cooked vegetables	229.2 (186.9)	107.6* (118.0)	-121.6 (196.0)	0.27	37	61	4
Raw vegetables	65.8 (45.1)	35.5* (38.4)	-30.3 (47.7)	0.42	50	66	3
Pizza, lasagna and quiche^b^	14.9 (26.7)	12.2 (21.0)	-2.7 (31.6)	0.24	38	.	.
Sandwich, burgers and kebab^b^	2.8 (16.0)	2.1 (7.1)	-0.6 (12.5)	0.41	86	.	.
Fish fingers/ breaded meat^b^	3.5 (8.7)	4.8 (12.4)	1.3 (13.9)	0.22	59	.	.
Sausages and other processed meat	33.7 (27.7)	23.6* (37.3)	-10.0 (40.0)	0.31	41	65	4
Poultry/rabbit	32.9 (28.5)	26.3* (36.4)	-6.6 (42.8)	0.24	43	61	5
Meat	52.2 (37.0)	37.1* (37.4)	-15.0 (46.9)	0.31	44	68	3
Offal^b^	3.4 (8.6)	2.8 (5.2)	-0.6 (9.7)	**0.10**	62	.	.
Eggs^b^	11.6 (14.4)	14.6* (13.2)	3.0 (16.5)	0.27	35	.	.
Fish^b^	46.1 (78.2)	16.0* (17.5)	-30.1 (78.1)	0.27	44	.	.
Seafood (excluding fish)^b^	9.6 (24.1)	3.3* (5.5)	-6.3 (23.8)	0.20	51	.	.
Milk^b^	60.6 (98.8)	118.6* (304.4)	58.0 (267.2)	0.77	65	.	.
Yoghurt, fromage blanc, cottage cheese	71.0 (64.2)	78.6 (74.3)	7.6 (63.7)	0.59	54	79	2
Cream dessert^b^	11.3 (25.9)	16.7 (31.6)	5.4 (31.4)	0.39	52	.	.
Cheese	33.0 (21.7)	30.2 (47.1)	-2.8 (47.5)	0.43	50	68	3
Butter, crème fraîche^b^	6.3 (7.0)	10.3* (14.8)	4.0 (11.4)	0.59	49	.	.
Margarine, mayonnaise^b^	3.7 (6.4)	5.0* (9.8)	1.3 (8.7)	0.54	62	.	.
Olive oil^b^	2.5 (3.7)	5.3* (7.0)	2.8 (6.4)	0.52	57	.	.
Rapeseed oil, walnut oil, mixed oil^b^	2.0 (3.0)	2.9 (4.7)	0.9 (4.7)	0.53	50	.	.
Sunflower oil, groundnut oil^b^	1.3 (2.5)	1.9 (4.5)	0.6 (4.0)	0.45	61	.	.
Salty snacks^b^	3.0 (6.7)	2.9 (6.7)	-0.1 (6.7)	0.35	57	.	.
Sweet snacks, chocolate and viennoiseries^b^	38.6 (37.1)	21.0* (44.5)	-17.5 (50.8)	0.35	48	.	.
Fruit	270.1 (157.8)	295.9 (268.7)	25.8 (251.3)	0.41	47	69	4
Water	1201.0 (481.9)	982.2* (679.6)	-218.8 (641.7)	0.42	54	72	3
Coffee	267.9 (192.6)	387.8* (472.7)	119.9 (425.2)	0.61	61	76	2
Tea and herb teas^b^	93.1 (183.9)	124.3* (280.4)	31.2 (160.2)	0.79	74	.	.
Fruit juice^b^	48.1 (79.6)	108.9* (294.4)	60.8 (272.9)	0.61	61	.	.
Sweet beverages^b^	19.9 (64.9)	97.8* (500.9)	77.8 (487.6)	0.40	71	.	.
Artificially-sweetened beverages^b^	18.6 (133.7)	72.1 (432.1)	53.5 (416.0)	0.48	89	.	.
Wine^b^	77.8 (125.7)	90.0 (156.7)	12.2 (105.1)	0.77	68	.	.
Other alcoholic beverages^b^	40.0 (81.7)	34.9 (94.4)	-5.1 (93.9)	0.47	54	.	.

*Significantly different from the value of the mean of the six 24-h recalls, according to Wilcoxon signed rank tests

^a^For food groups with a proportion of non-consumers > 20%^b^, tertiles and quintiles classifications were not performed. Instead, participants were classified as follows: class = 1 for null consumption; class = 2 for consumption below or equal to the median value in consumers, class = 3 for consumption above the median value in consumers

^b^These food groups have a large proportion of non-consumers (>20%)

The statistical tests provided *p*-values <0.05 for each unadjusted Spearman correlation coefficient, except for those in bold

Unadjusted Spearman coefficients ranged from 0.05 (legumes) to 0.79 (tea and herb teas), the median value being 0.40. 13 food groups had correlation coefficients below 0.3.

The median proportion of participants classified in the same and adjacent quintiles of food group consumption by the SFFQ2, as well as by the mean of the six 24-h recalls, was 68.0%. The median proportion of misclassified participants was 3.0%. The median proportion of participants classified in the same tertile was 51.0%.

#### Nutrients

Mean macronutrient intakes estimated using the SFFQ2 were not statistically significantly different from those estimated using the six 24-h recalls, except for protein, dietary fibre and cholesterol intakes which were underestimated by the SFFQ (Table 3). When micronutrient intakes estimated using the SFFQ were different from those estimated using the six 24-h recalls (*p* value < 0.05), they were underestimated by the SFFQ, except for retinol and manganese intakes.

**Table 3:** Relative validity of the SFFQ for nutrients (*n* = 127)

	Daily intakes			Pearson correlation coefficients			Cross-classification of nutrient distribution	
	24-hour recalls	SFFQ 2	SFFQ2-24-hour recalls	SFFQ2 *vs.* means of six 24-hour recalls				
	Mean (SD)		Means of differences (SD)	Unadjusted	Energy-adjusted^a^	De-attenuated^b^	% of subjects classified in same or adjacent quintile	% of misclassified subjects
Energy (kcal)	1758.7 (490.6)	1748.1 (722.8)	-10.5 (669.5)	0.40	.	.	67	4
Protein (g)	75.1 (19.9)	69.7* (28.0)	-5.5 (27.1)	0.39	0.39	0.46	68	2
Carbohydrates (g)	196.3 (63.7)	201.5 (102.3)	5.2 (98.7)	0.36	0.39	0.42	65	5
Fat (g)	61.5 (19.6)	60.0 (28.3)	-1.4 (26.8)	0.44	0.34	0.40	66	1
SFA (g)	24.1 (8.6)	23.6 (13.6)	-0.5 (12.7)	0.47	0.39	0.47	66	1
MUFA (g)	20.4 (7.0)	21.5 (10.1)	1.1 (9.5)	0.43	0.31	0.39	69	2
PUFA (g)	9.2 (4.3)	8.9 (4.9)	-0.4 (5.3)	0.41	0.40	0.46	67	1
Cholesterol (mg)	263.5 (98.5)	234.2* (109.8)	-29.3 (107.5)	0.52	0.44	0.59	68	1
Sugars (g)	75.6 (29.9)	78.3 (71.7)	2.8 (65.9)	0.32	0.32	0.34	65	7
Fibre (g)	19.5 (6.3)	18.4* (8.4)	-1.1 (8.9)	0.35	0.40	0.44	67	5
Alcohol (g)^c^	10.5 (15.5)	10.7 (17.3)	0.2 (12.4)	0.76			87	0
Retinol (μg)	17.9 (66.0)	496.0* (429.8)	478.1 (428.1)	**0.42**	**0.43**	0.44	39	20
Carotene (μg)	3372.7 (1627.2)	2318.8* (1604.1)	-1054.0 (1888.6)	0.21	0.21	0.29	66	5
Vitamin B1 (mg)	1.1 (0.3)	1.0* (0.5)	-0.1 (0.5)	0.32	0.27	0.33	61	4
Vitamin B2 (mg)	1.4 (0.5)	1.4 (0.8)	0.0 (0.7)	0.36	0.41	0.47	66	5
Vitamin B3 (mg)	16.6 (5.0)	15.6 (6.8)	-1.0 (7.1)	0.27	0.22	0.29	62	3
Vitamin B5 (mg)	4.5 (1.3)	4.6 (2.3)	0.1 (2.2)	0.27	**0.16**	0.18	59	5
Vitamin B6 (mg)	1.7 (0.6)	1.5* (0.6)	-0.2 (0.8)	0.26	0.31	0.35	61	5
Vitamin B9	281.7 (90.4)	281.1 (134.2)	-0.6 (139.0)	0.29	0.32	0.37	60	5
Vitamin B12 (μg)^c^	10.3 (19.2)	5.9 (3.6)	-4.4 (19.2)	0.31			60	3
Vitamin C (mg)	112.2 (53.2)	107.9* (113.7)	-4.3 (112.8)	0.20	0.19	0.22	63	7
Vitamin D (μg)	9.1 (1.5)	1.9* (1.0)	-7.2 (1.7)	0.21	0.18	0.26	59	3
Vitamin E (mg)	9.3 (3.9)	10.4 (5.8)	1.1 (5.6)	0.47	0.45	0.52	73	1
Calcium (mg)	1171.0 (338.7)	956.9* (616.5)	-214.1 (560.6)	0.41	0.39	0.43	63	4
Iron (mg)	10.8 (4.0)	10.2 (4.5)	-0.6 (5.1)	0.28	**0.17**	0.20	61	3
Magnesium (mg)	307.5 (90.2)	329.3 (152.4)	21.8 (138.9)	0.31	0.27	0.29	60	4
Zinc (mg)	10.3 (4.1)	8.8* (3.6)	-1.5 (4.5)	0.33	0.19	0.24	66	5
Phosphorus (mg)	1083.5 (267.9)	1067.8 (492.5)	-15.7 (457.0)	0.36	0.34	0.39	69	6
Manganese (mg)	3.3 (1.7)	11.0* (5.1)	7.7 (4.9)	0.20	**0.11**	0.11	54	8
Potassium (mg)	2959.8 (793.5)	2983.1 (1547.5)	23.3 (1491.1)	0.31	0.29	0.32	66	6
Iodine (μg)	121.3 (62.3)	108.6* (52.5)	-12.7 (68.1)	0.32	0.22	0.27	65	5
Copper (mg)	2.3 (1.2)	2.3 (1.4)	0.0 (1.6)	0.41	0.37	0.41	69	2
Sodium (mg)	5058.4 (1734.1)	2319.3* (1066.2)	-2739.1 (1875.6)	0.22	**0.10**	0.12	64	6
Water (g)	2681.7 (604.0)	2674.0 (1454.4)	-7.7 (1458.4)	0.11	0.10	0.10	65	9

*SFA* Saturated fatty acids, *MUFA* Monounsaturated fatty acids, *PUFA* Polyunsaturated fatty acids

Means and cross-classification were computed on crudes variables. All variables were log transformed before computing Pearson correlation coefficients, to improve normality

*Significantly different from the value of the mean of the six 24-h recalls, according to Wilcoxon signed rank tests

^a^Energy adjustment according to the residual method

^b^De-attenuated and energy-adjusted Pearson’s correlation coefficient (corrected for within-person variation in six 24- h recalls)

^c^Spearman correlation coefficients were performed because normality was not respected

The statistical tests provided *p*-values <0.05 for each Pearson correlation coefficient, except for those in bold

Unadjusted correlation coefficients ranged from 0.11 (water) to 0.76 (alcohol), with the median value being 0.32. De-attenuation improved energy-adjusted correlation coefficients. De-attenuated CC ranged from 0.10 (water) to 0.59 (cholesterol), with the median value being 0.35. Correlation coefficients for nutrients of interest for CKD patients, including protein, calcium, phosphorus, and potassium ranged from 0.32 (potassium) to 0.46 (protein). CC for carbohydrates and lipids were 0.42 and 0.40 respectively. A total of 12 nutrients (carotene, vitamins B3, B5, C, D, iron, magnesium, zinc, manganese, iodine, sodium and water) had correlation coefficients lower than 0.3.

The median of percentages of participants classified in the same and adjacent quintiles of nutrient intakes by the SFFQ2, as well as by the mean of the six 24-h recalls, was 65.0%. The median proportion of misclassified participants was 4.5%. Regarding nutrients of interest for CKD patients, percentages of participants classified in the same and adjacent quintiles ranged from 63% (calcium) to 69% (phosphorus) and the median proportion of misclassified participants ranged from 1% (lipids) to 6% (sodium, phosphorus and potassium).

**Fig. 1:**
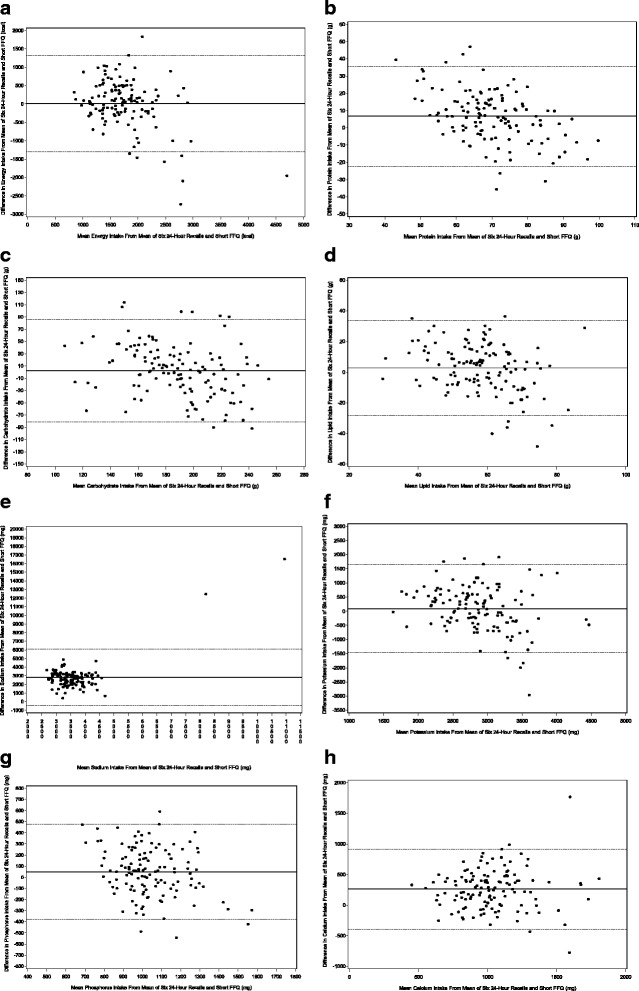
Bland-Altman plots related to macronutrients and micronutrients of interest in CKD population. Difference in the daily intake of some energy-adjusted macronutrients and micronutrients [a) energy, b) protein, c) carbohydrate, d) lipid, e) sodium, f) potassium, g) phosphorus, h) calcium] derived from the six 24-h recalls and the food frequency questionnaire (FFQ2) plotted against the corresponding mean energy-adjusted daily intakes derived from the two methods. Solid lines represent mean difference, and dashed lines show lower and upper 95% limits of agreement (mean ± 1.96 SD) (*n* = 127)

The Bland-Altman plot analysis graphs show good agreement between the two methods of estimation, across the range of intake, for macronutrients and nutrients of interest for CKD patients (Fig. 1). The mean difference between methods was near zero for all levels of intake, except for sodium and calcium. Across the range of intakes, sodium was systematically underestimated by the SFFQ2, which was consistent with the results displayed in Table 3. The percentage of points that were outside the limits of agreement ranged from 0.8% (vitamin B6) to 8.7% (vitamine B5), with a median value of 4.7%, which is theoretically the percentage of values outside the mean ± 1.96 SD. Finally, the agreement did not differ between high and low intakes.

### Reproducibility

#### Food groups

Absolute daily intakes of food groups were mostly comparable between the two SFFQs, except for ‘white bread and substitutes’, ‘sandwiches, burgers and kebabs’, ^‘^offal’, ‘eggs’, ‘fruit’, and ‘artificially-sweetened beverages’ that showed a decrease between SFFQ1 and SFFQ2 (Table 4).

**Table 4:** Reproducibility of food group consumptions of the SFFQ (*n* = 201)

	Daily intakes			Spearman correlation coefficients		Cross-classification of food groups distribution		
	SFFQ 1	SFFQ 2	SFFQ2-SFFQ1	SFFQ2 vs.*S*FFQ1				
	Mean in grams (foods) or ml (beverages) (SD)		Means of differences, in grams (foods) or ml (beverages) (SD)	Unadjusted	ICC	% of subjects classified in same tertile^a^	% of subjects classified in same or adjacent quintile	% of misclassified subjects
Whole-grain bread and substitutes^b^	33.2 (70.5)	29.4 (50.7)	−3.8 (66.8)	0.47	0.41	63		
White bread and substitutes	104.6 (103.5)	86.4* (91.3)	−18.1 (112.0)	0.40	0.34	52	73	1
Breakfast cereals^b^	2.4 (9.2)	2.0 (7.5)	−0.4 (7.9)	0.63	0.55	89		
Whole-grain pasta, rice and wheat^b^	23.9 (48.5)	23.4 (38.6)	−0.5 (51.2)	0.43	0.32	57		
White pasta, rice and wheat	53.3 (63.4)	49.7 (57.6)	−3.6 (66.1)	0.54	0.40	54	74	1
Legumes^b^	33.9 (70.5)	27.5 (50.0)	−6.5 (84.7)	0.46	0.04	58		
French fries and other fried tubers^b^	12.0 (15.6)	11.1 (15.1)	−0.9 (19.2)	0.49	0.22	58		
Potatoes and other tubers (not fried)	55.5 (58.3)	54.8 (54.2)	−0.6 (61.1)	0.46	0.41	59	69	2
Cooked vegetables	108.9 (120.2)	102.4 (112.5)	−6.5 (97.7)	0.64	0.65	62	80	1
Raw vegetables	40.8 (40.6)	38.1 (45.8)	−2.6 (42.0)	0.54	0.53	63	74	1
Pizza, lasagna and quiche^b^	13.2 (19.2)	12.7 (19.0)	−0.5 (20.2)	0.55	0.44	56		
Sandwich, burgers and kebab^b^	7.0 (21.3)	4.2* (11.5)	−2.8 (19.6)	0.70	0.35	82		
Fish fingers/breaded meat^b^	8.4 (25.5)	5.9 (13.4)	−2.6 (28.5)	0.47	0.02	64		
Sausages and other processed meat^b^	20.2 (26.7)	23.5 (39.0)	3.3 (37.7)	0.63	0.36	63		
Poultry/rabbit	32.4 (53.1)	30.8 (50.8)	−1.6 (41.0)	0.50	0.69	51	74	2
Meat	45.3 (47.8)	39.2 (40.2)	−6.1 (49.6)	0.50	0.37	61	75	5
Offal^b^	4.2 (8.0)	3.5* (12.3)	−0.7 (13.7)	0.47	0.13	69		
Eggs	21.1 (26.5)	16.5* (17.9)	−4.5 (24.5)	0.55	0.41	49	75	1
Fish	16.4 (15.4)	16.6 (17.8)	0.1 (18.4)	0.49	0.39	50	73	1
Seafood (excluding fish)^b^	5.2 (11.8)	3.2 (5.3)	−1.9 (11.7)	0.57	0.18	64		
Milk^b^	99.0 (169.4)	96.3 (248.8)	−2.8 (196.4)	0.77	0.57	81		
Yoghurt, fromage blanc, cottage cheese	93.8 (99.0)	84.0 (82.1)	−9.8 (88.0)	0.63	0.53	70	84	1
Cream dessert^b^	16.7 (34.8)	20.2 (42.9)	3.5 (40.7)	0.72	0.46	72		
Cheese	26.1 (25.2)	26.6 (40.0)	0.5 (40.4)	0.54	0.27	60	77	3
Butter, crème fraîche^b^	11.9 (16.3)	11.6 (18.7)	−0.4 (18.9)	0.58	0.42	56		
Margarine, mayonnaise^b^	5.4 (11.5)	4.7 (10.3)	−0.6 (8.0)	0.62	0.73	63		
Olive oil^b^	7.0 (9.9)	5.6 (7.2)	−1.4 (8.6)	0.63	0.51	66		
Rapeseed oil, walnut oil, mixed oil^b^	3.8 (7.0)	3.5 (6.5)	−0.4 (7.9)	0.61	0.32	64		
Sunflower oil, groundnut oil^b^	2.3 (5.1)	1.8 (4.5)	−0.4 (3.5)	0.53	0.73	66		
Salty snacks^b^	3.1 (6.3)	2.8 (5.2)	−0.3 (6.4)	0.54	0.38	61		
Sweet snacks, chocolate and viennoiseries^b^	27.8 (82.6)	19.5 (38.6)	−8.4 (75.3)	0.53	0.32	55		
Fruit	313.7 (272.7)	292.7* (283.1)	−21.1 (319.4)	0.51	0.34	59	75	2
Water	934.8 (625.7)	971.1 (672.8)	36.4 (670.8)	0.46	0.47	55	75	3
Coffee	381.7 (443.6)	366.6 (428.5)	−15.1 (406.1)	0.71	0.57	64	83	1
Tea and herb teas^b^	137.2 (311.7)	113.0 (250.7)	−24.2 (260.4)	0.79	0.58	76		
Fruit juice^b^	93.2 (177.0)	103.4 (246.3)	10.2 (256.6)	0.59	0.28	63		
Sweet beverages^b^	77.0 (242.4)	98.1 (441.1)	21.0 (404.4)	0.51	0.35	75		
Artificially-sweetened beverages^b^	90.2 (379.3)	71.7* (363.3)	−18.5 (427.1)	0.59	0.34	81		
Wine^b^	94.3 (197.5)	81.4 (143.2)	−12.9 (127.5)	0.86	0.73	84		
Other alcoholic beverages^b^	45.2 (180.3)	30.4 (78.5)	-14.8 (155.3)	0.71	0.38	71		

*ICC* Intraclass correlation coefficient

*Significantly different from the value of the SFFQ1, according to Wilcoxon signed rank tests

^a^For food groups with a proportion of non-consumers > 20%^b^, tertiles and quintiles classifications were not performed. Instead, participants were classified as follows: class = 1 for null consumption; class = 2 for consumption below or equal to the median value in consumers, class = 3 for consumption above the median value in consumers

^b^These food groups have a large number of non-consumers (>20%)

The statistical tests provided *p*-values <0.05 for each unadjusted Spearman correlation coefficient

Unadjusted Spearman correlation coefficients ranged from 0.40 (white bread and substitutes) to 0.86 (wine), with the median value being 0.54. Intraclass correlation coefficients ranged from 0.02 (‘Fish fingers/breaded meat’) to 0.73 (‘wine’, ‘sunflower oil, groundnut oil’ and ‘margarine, mayonnaise’), with the median value being 0.40. Seven food groups (‘legumes’, ‘French Fries and other fried tubers’, ‘fish fingers/breaded meat’, ‘seafood (excluding fish)’, ‘offal’, ‘cheese’ and ‘fruit juice’) had intraclass correlation coefficients lower than 0.3.

The median of percentages of subjects classified in the same and adjacent quintiles of food group consumption by both SFFQs was 75.0%. The median proportion of misclassified participants was 1.0%. The median of percentages of subjects classified in the same tertile was 63.0%.

#### Nutrients

Absolute daily intake of energy and nutrients were comparable between the two SFFQs, although all nutrient intakes showed a significant slight decrease between SFFQ1 and SFFQ2 (Table 5).

**Table 5:** Reproducibility of nutrient intakes of the SFFQ (*n* = 201)

	Daily intakes			Pearson correlation coefficients			Cross-classification of nutrient distribution	
	SFFQ 1	SFFQ 2	SFFQ2-SFFQ1	SFFQ2 vs.*S*FFQ1				
	Mean (SD)		Means of differences (SD)	Unadjusted	Energy-adjusted^a^	ICC	% of subjects classified in same or adjacent quintiles	% of misclassified subjects
Energy (kcal)	1938.8 (808.9)	1745.6* (681.0)	−193.3 (801.1)	0.50		0.43	75	2
Protein (g)	77.9 (33.8)	70.8* (29.4)	−7.1 (32.7)	0.51	0.51	0.47	71	2
Carbohydrates (g)	221.7 (110.1)	199.5* (94.9)	−22.2 (118.9)	0.42	0.39	0.33	74	4
Fat (g)	67.1 (34.0)	61.0* (28.5)	−6.2 (30.7)	0.58	0.49	0.52	76	1
SFA (g)	25.3 (14.4)	23.5* (13.5)	−1.7 (13.8)	0.59	0.49	0.51	76	2
MUFA (g)	24.7 (13.2)	22.1* (10.6)	−2.6 (11.5)	0.59	0.53	0.54	75	1
PUFA (g)	10.2 (6.0)	9.0* (5.4)	−1.1 (5.4)	0.56	0.57	0.55	77	2
Cholesterol (mg)	278.7 (174.1)	248.0* (134.8)	−30.7 (157.3)	0.54	0.48	0.49	79	1
Sugars (g)	80.3 (51.7)	77.0* (63.7)	−3.3 (65.6)	0.54	0.48	0.36	79	2
Fibre (g)	20.5 (10.1)	18.3* (8.7)	−2.1 (10.9)	0.39	0.43	0.33	72	3
Alcohol (g)^b^	11.8 (23.2)	9.6 (15.5)	−2.2 (16.3)	0.90		0.66	97	0
Retinol (μg)	572.3 (505.2)	534.5* (750.5)	−37.8 (820.0)	0.48	0.46	0.18	81	4
Carotene (μg)	2467.3 (1742.6)	2308.5 (1618.7)	−158.8 (1474.7)	0.58	0.56	0.62	79	2
Vitamin B1 (mg)	1.0 (0.4)	1.0* (0.5)	−0.1 (0.5)	0.49	0.35	0.48	67	2
Vitamin B2 (mg)	1.6 (0.7)	1.4* (0.7)	−0.1 (0.7)	0.54	0.41	0.53	76	2
Vitamin B3 (mg)	17.6 (8.3)	16.2* (7.8)	−1.4 (8.2	0.46	0.44	0.48	72	4
Vitamin B5 (mg)	5.2 (2.1)	4.7* (2.2)	−0.5 (2.2)	0.49	0.37	0.49	73	3
Vitamin B6 (mg)	1.6 (0.7)	1.5* (0.6)	−0.1 (0.7)	0.47	0.41	0.45	73	4
Vitamin B9	307.4 (130.2)	282.2* (131.8)	−25.3 (138.5)	0.47	0.41	0.44	73	3
Vitamin B12 (μg)	7.2 (5.2)	6.3* (4.9)	−0.9 (6.3)	0.48	0.45	0.23	79	2
Vitamin C (mg)	107.0 (72.6)	105.6 (98.7)	−1.4 (100.7)	0.47	0.43	0.32	76	2
Vitamin D (μg)	2.1 (1.1)	2.0 (1.0)	−0.1 (1.2)	0.53	0.55	0.38	75	2
Vitamin E (mg)	12.2 (7.5)	10.7* (6.6)	−1.5 (6.5)	0.59	0.60	0.58	78	1
Calcium (mg)	962.2 (413.4)	908.0* (527.2)	−54.3 (456.2)	0.61	0.55	0.54	81	0
Iron (mg)	11.6 (5.6)	10.2* (4.5)	−1.3 (5.5)	0.46	0.37	0.42	71	1
Magnesium (mg)	353.2 (140.2)	325.8* (141.1)	−27.4 (146.4)	0.49	0.46	0.46	70	3
Zinc (mg)	9.8 (4.5)	8.8* (3.6)	−1.0 (4.4)	0.52	0.49	0.42	74	2
Phosphorus (mg)	1155.5 (471.3)	1058.8* (450.7)	−96.7 (473.2)	0.55	0.52	0.47	79	2
Manganese (mg)	11.8 (4.7)	10.9* (4.7)	−0.9 (4.9)	0.49	0.46	0.46	71	3
Potassium (mg)	3147.4 (1217.2)	2934.6* (1403.5)	−212.7 (1388.9)	0.50	0.41	0.44	74	1
Iodine (μg)	120.5 (58.0)	108.1* (47.6)	−12.4 (52.5)	0.58	0.57	0.51	79	2
Copper (mg)	2.5 (1.4)	2.3* (1.4)	−0.2 (1.5)	0.52	0.52	0.44	70	1
Sodium (mg)	2552.0 (1164.6)	2290.1* (1057.8)	−261.9 (1227.0)	0.42	0.32	0.39	67	3
Water (g)	2688.6 (1222.6)	2608.9 (1287.2)	−79.7 (1299.2)	0.53	0.44	0.46	70	2

Means and cross-classification were computed on crudes variables. All variables were log transformed before computing Pearson correlation coefficients, to improve normality

^a^ Energy adjustment according to the residual method

^b^ Spearman correlation coefficients were performed because normality was not respected

*Significantly different from the value of the SFFQ1, according to Wilcoxon signed rank tests

The statistical tests provided *p*-values <0.05 for each Pearson correlation coefficient

Crude correlation coefficients ranged from 0.39 (fibre) to 0.90 (alcohol), with the median value being 0.52. Energy-adjusted Pearson correlation coefficients ranged from 0.32 (sodium) to 0.60 (vitamin E), with the median value being 0.46. Intraclass correlation coefficients ranged from 0.18 (retinol) to 0.66 (alcohol), with the median value being 0.46. ICC for five of the most important nutrients of interest for CKD patients (i.e. protein, calcium, phosphorus, potassium and sodium) ranged from 0.39 (sodium) to 0.54 (calcium). ICC were 0.33 and 0.52 for carbohydrates and lipids, respectively. Two nutrients (retinol and vitamin B12) had intraclass correlation coefficients lower than 0.3.

The median proportion of subjects classified in the same and adjacent quintile of nutrient intakes by both SFFQs was 75.0%. The median proportion of misclassified participants was 2.0%. Regarding the nutrients of interest for CKD patients, the proportion of subjects classified in the same and adjacent quintile ranged from 67% (sodium) to 81% (calcium). The median proportion of misclassified participants ranged from 0% (calcium) to 3% (sodium).

## Discussion

The present study investigated the validity and reproducibility of a new SFFQ, customized for patients with CKD. This SFFQ was designed to estimate energy intake and to rank participants according to their dietary and nutrient intakes. The overall results indicate acceptable relative validity (for nutrient intakes, median correlation coefficient = 0.35 and median proportion of subjects classified in the same or adjacent quintiles by the SFFQ2 and the 24-h recalls = 65.0%) and good reproducibility (for nutrient intakes, median correlation coefficient = 0.46 and median proportion of subjects classified in the same or adjacent quintiles = 75.0%). Our tool demonstrated an acceptable ability to rank participants for most nutrients (including nutrients of interest for CKD patients: protein, calcium, phosphorus, potassium, sodium and carbohydrates and lipids) and food groups making it sufficiently informative to evaluate associations with health outcomes and adjust for nutritional intake in epidemiological studies [37, 41]. It can also be used to derive dietary patterns using collected food data.

In this study, six 24-h recalls were used as the reference method for determining the validity of the SFFQ. Repeated dietary recalls, despite their limitations, are one of the most used reference methods for validation studies of FFQs [42–44]. Even if there is some evidence that increasing the number of recording days in the reference method improves the apparent validity of a questionnaire [45], the optimal number of days of dietary recording has been discussed in the literature. Some authors have concluded that 8 days were necessary to accurately assess most nutrient intakes [46], but other authors have stated that the optimal study design would rarely require more than four or 5 days of dietary recording [47].

All French studies with data on food groups had similar ranges of unadjusted coefficients for validity [29–32]. 13 food groups had correlation coefficients below 0.3. 10 of them were rarely consumed foods (e.g. offal or legumes) which could explain why we observed such low CC for these foods groups. Such findings on rarely consumed foods have been previously reported [30–32]. Some food groups (e.g. vegetables, or alcoholic beverages) may have been over- or under-reported because of social approval or social desirability: healthy *vs*. unhealthy image of some food groups [41, 48, 49] and supposed consumption restriction of some of them because of CKD context (e.g. meat, poultry, fish, eggs, milk and dairy products, whole-grain foods, legumes and some vegetables [8, 50]).

For nutrients, the correlation coefficients were similar to those obtained in an international review for energy, fat and alcohol but were lower for protein, carbohydrates, calcium, vitamin C and dietary fiber [43]. Differences in the means of sodium, as estimated by both methods, were high. Even though a question about extra-salt added after food preparation was asked in the CKD-REIN-specific part of the questionnaire, it was still difficult to estimate its intake. 12 nutrients (carotene, vitamins B3, B5, C, D, iron, magnesium, zinc, manganese, iodine, sodium and water) had correlation coefficients lower than 0.3. Among those nutrients, sodium is of importance in CKD context. Even if CC was low for sodium, the SFFQ showed acceptable validity to rank people according to sodium intake (64% of people classified in the same or adjacent quintile when comparing sodium intake with the SFFQ and the 6 24-h recalls).

Some research teams, including the Deschamps et al. one [30], have previously developed long FFQs [29, 31, 32]. We were expecting lower correlation coefficients in our study in comparison with those studies, as increasing the number of food items should enable to better capture food intakes and therefore nutrient intakes. But, our results were similar to most of the previous work, except the FFQ obtained by Deschamps et al. [30] Regarding macronutrients [31] and other nutrients [29, 32], we obtained similar or higher correlation coefficients than other studies using longer questionnaires.

We obtained lower correlation coefficients than Barrat et al. [23] and Vercambre et al. [21] who developed SFFQs; probably due to the one-week time frame of the questionnaire developed, and the design of the validity study, respectively.

Adjustment for energy led to a decrease in correlation coefficients for most nutrients, which has been frequently reported and would be more related to a variability due to systematic errors of under/overestimation than to energy intake [30, 37]. De-attenuation led to improvement of coefficients as expected, but was less pronounced than in another study [32], probably due to the lower number of recalls used here (6 *vs*. 12).

Agreement in classification was acceptable, including for the nutrients of interest in CKD. Our results were close to the recommended 70% [51] and similar or slightly lower than those reported in other studies [21, 23, 30, 32]. The lowest level of agreement was observed for retinol (39% of subjects within the same or adjacent quintiles). One of the main sources of retinol is offal, which was rarely consumed and whose consumption was probably difficult to evaluate with only six 24-h recalls.

To study the SFFQ reproducibility, we adopted a one-year time interval which is long but frequently used and reported as acceptable [42, 43, 52]. When studying the reproducibility of a FFQ, the time frame between the two administrations of the tool has to be sufficiently long to prevent participants from remembering and repeating their responses. However, when a longer interval is used, true changes in dietary habits as well as variation in response contribute to reduced reproducibility [53]. Therefore the observed reproducibility here may be lower than the true value.

Our study showed acceptable reproducibility for most foods (range: 0.40–0.86) and nutrients (ranges: 0.32–0.60 and 0.32–0.53 for overall nutrients, and the CKD nutrients of interest respectively), with the best reproducibility observed for wine and vitamin E. Our findings were comparable to prior reported correlation coefficients for reproducibility [54].

According to a review, correlation coefficients of 0.5 to 0.7 between two administrations are commonly reported [42]. In our study, 72% and 59% of the food groups and nutrients studied had correlation coefficients ≥0.5. The proportion of correlation coefficients <0.5 may be due to the time interval between the two administrations. Patients with CKD may be at higher risk to modify their diet over a one-year period because of disease complications. The seven food groups with the lowest ICC (ICC < 0.3) corresponded to food groups which are rarely consumed in this population (i.e. ‘cheese’).

Despite CKD context, agreement in classification was very good (median of 75% and 74% for overall foods and nutrients, and the CKD nutrients of interest respectively).

In comparison with other FFQs used in a CKD context [55–59], we obtained similar results in terms of validity and reproducibility. When considering validity, the FFQ used by Mirmiran et al. [12] had crude correlation coefficients that ranged from 0.33 (legumes and nuts) to 0.79 (tea and coffee) [12, 55]. When the FFQ used by Lew et al. [11] was validated, energy and de-attenuated CC for nutrients ranged from 0.24 to 0.79 [58]. The SFFQ used by Domingos et al. [16] had ICC in the validation study that ranged between 0.17 (selenium) to 0.66 (calcium) [59]. Reproducibility results of the FFQ used by Diaz-Lopez et al. [13] were slightly higher than ours [57], but participants were specifically asked not to modify their dietary habits during the study’s 1-year duration. In another study, reproducibility Spearman CC obtained for food groups were much lower than ours (range: 0.19–0.67; median = 0.35 vs. range: 0.40–0.86; median = 0.54) but reproducibility was evaluated over an 8 year interval [55].

### Strengths and limitations

The current work has some limitations, inherent to nutritional epidemiology. Learning effect was not measurable. Authors that compared both FFQs with 24-h recalls found that the second FFQ was more valid than the first. However, with the completion of the six 24-h recalls during the year, participants may pay more attention to their diet and are therefore in distinct conditions when fulfilling the second FFQ.

Furthermore, dietary intake cannot be estimated without error [60]. Comparison of energy intake(EI) with minimal energy requirements (basal metabolic rate- BMR) provides an indirect indication of bias. In our study, taking into account the cutoff value of 1.55 for a minimal activity level [27], EI/BMR estimates suggested underreporting by both SFFQ and 24-h recalls(Median < 1.55) (see additional file 4). We did not observe any major differences between the two methods. Under or overestimation are not necessarily problematic in epidemiological studies if ranking of people according to their dietary intake is valid [37].

Our work also has several strengths. In comparison to other validity and reproducibility studies, we worked on data from a large sample [42] and managed to maintain high response levels all over the one-year interval despite working with an aging population, with CKD.

Several CKD cohorts exist on an international level [61]. However few of them assess diet despite the impact of diet in CKD management. We developed a rapid tool to assess energy intake in CKD patients. It showed acceptable validity and reproducibility to rank people according to their food group and nutrient intakes, including nutrients of interest for CKD patients.

The tool we developed was easy to complete and not time consuming. With the portion size photos directly integrated into the questionnaire, it was easy for participants to estimate the amounts of food consumed.

Here, we present validity results for the SFFQ in a CKD sample. Further validity studies will be conducted in other population subgroups. One of the main strengths of the consortium work is that we will have a unique tool (due to the shared 40 items in the first part of the SFFQ), useful for several population subgroups. Diet and dietary patterns will therefore be comparable between studies and a standardized dietary assessment will be available for epidemiological and clinical research.

The methodology we used to obtain a standardized SFFQ could be adapted for other European and worldwide countries in order to foster international studies of nutrition in patients with CKD. Developing a smartphone app of the SFFQ is the next step. This type of tool may further be used in clinical routine to monitor patient nutrient intakes and provide them instantaneous feedback and recommendations about their diet.

## Conclusions

For most food groups and nutrients, including nutrients of interest in CKD, the SFFQ showed acceptable validity and reproducibility in a sample of patients with CKD. Before being administered to a large sample, some minor modifications without substantial impact could be done to the questionnaire to improve its validity. Whole-grain and white bread, and whole-grain and white pasta could be grouped together. A question about added sugar and artificial sweeteners to hot beverages could also be added to the questionnaire, to better estimate sugar intakes.

## Additional files

Additional file 1: Flow diagram. (DOCX 85 kb)

Additional file 2: SFFQ items used to obtain nutritional data (*n* = 49). (DOCX 20 kb)

Additional file 3: Extract from the questionnaire. (DOCX 331 kb)

Additional file 4: Distribution of EI/BMR according to dietary method and reproducibility/validity study. (DOCX 16 kb)

## Abbreviations

BMR: Basal Metabolic Rate
CC: Correlation Coefficient
CKD: Chronic Kidney Disease
CKD-REIN: CKD-Renal Epidemiology and Information Network
DPI: Dietary Protein Intake
ESRD: End-Stage Renal Disease
FFQ: Food Frequency Questionnaire
GFR: Glomerular Filtration Rate
ICC: Intraclass Correlation Coefficient
SFFQ: Short Food Frequency Questionnaire
