# Supplementary material for: Validity and reproducibility of a short food frequency questionnaire among patients with chronic kidney disease
Source: BMC Nephrol. 2017 Sep 15;18:297. doi: 10.1186/s12882-017-0695-2 (PMC5599889; doi:10.1186/s12882-017-0695-2)
Supplement: Supplementary file 1 — Flow diagram. (DOCX 85 kb) [file 12882_2017_695_MOESM1_ESM.docx]

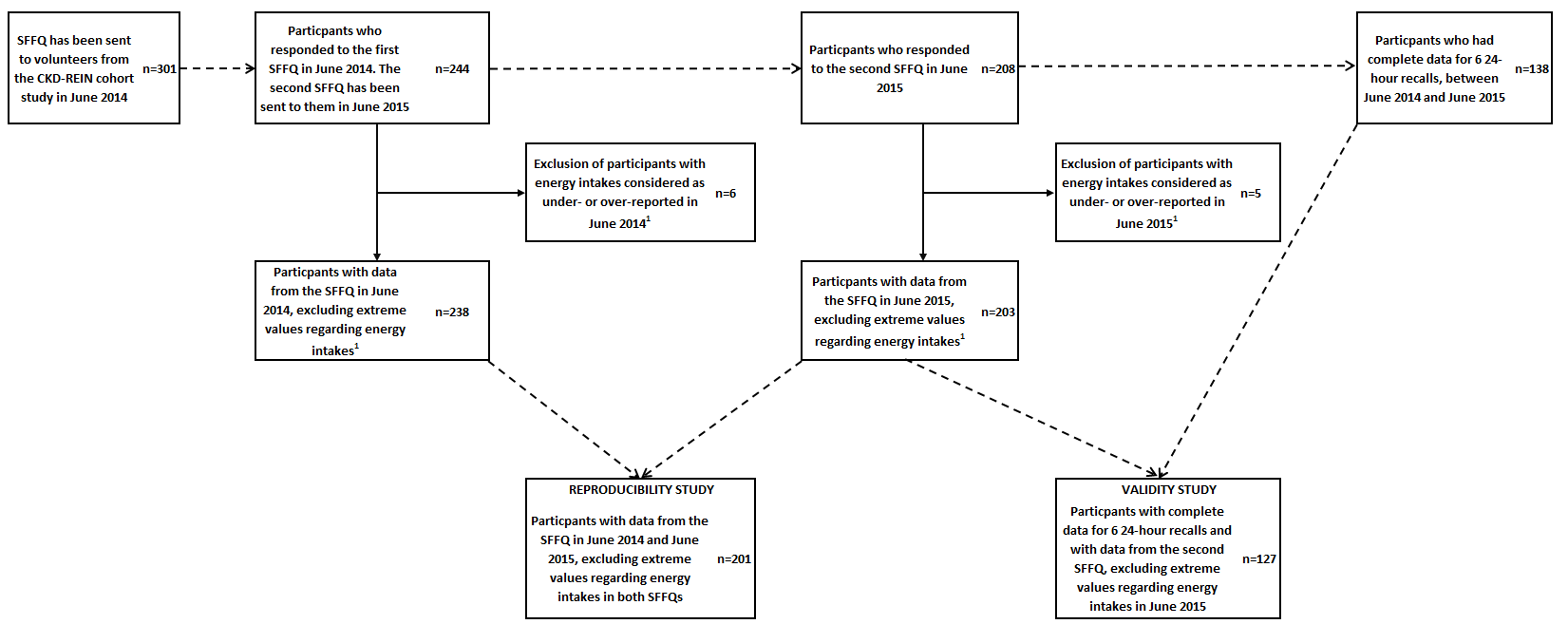


^1^ Participants who under- or over- reported energy intake in one of the SFFQs were excluded: they were in the top and bottom 1% of the energy intake to energy requirement ratio distribution. Energy requirement was calculated as follows: Basal Metabolic Rate (BMR)* Physical Activity Level (the cutoff value of 1.55 was chosen). BMR was computed on the basis of gender, age, height and weight, using the Schofield formula.
