# Supplementary material for: Validity and reproducibility of a short food frequency questionnaire among patients with chronic kidney disease
Source: BMC Nephrol. 2017 Sep 15;18:297. doi: 10.1186/s12882-017-0695-2 (PMC5599889; doi:10.1186/s12882-017-0695-2)
Supplement: Supplementary file 2 — SFFQ items used to obtain nutritional data (n = 49). (DOCX 20 kb) [file 12882_2017_695_MOESM2_ESM.docx]

**Additional file 2:** SFFQ items used to obtain nutritional data (n=49).

- 40 food group items were included in the first part of the questionnaire (NB: The first part of the questionnaire will be used to assess overall diet in different population subgroups included in the consortium):

| 1. Whole-grain bread and substitutes 2. White bread and substitutes 3. Breakfast cereals 4. Whole-grain pasta, rice and wheat 5. White pasta, rice and wheat 6. Legumes 7. French fries and other fried tubers 8. Potatoes and other tubers (not fried) 9. Cooked vegetables 10. Raw vegetables 11. Pizza, lasagna and quiche 12. Sandwiches, burgers and kebabs 13. Fish fingers/ breaded meat 14. Sausages and other processed meat 15. Poultry/rabbit 16. Meat 17. Offal 18. Eggs 19. Fish 20. Seafood (excluding fish) | 1. Milk 2. Yoghurt, fromage blanc, cottage cheese 3. Cream dessert 4. Cheese 5. Butter, sour cream 6. Margarine, mayonnaise 7. Olive oil 8. Rapeseed oil, walnut oil, mixed oil 9. Sunflower oil, groundnut oil 10. Salty snacks 11. Sweet cakes and snacks, chocolate and pastries 12. Fruit 13. Water 14. Coffee 15. Tea and herb teas 16. Fruit juice 17. Sweet beverages 18. Artificially-sweetened beverages 19. Wine 20. Other alcoholic beverages |
| --- | --- |

- 9 questions were included into the second part of the questionnaire (NB: These 9 nine questions are specific to CKD context):

1- How often do you add extra-salt? (Always, Really often, Quite often, Rarely, Never)

2- During last year, how many times did you eat processed food? (Never or less than once a month, *x* times a day, *x* times a week or *x* times a month)

- 7 questions provided more detailed information on some food group items asked in the first part of the questionnaire. Study participants were asked to score their relative consumption frequency for each single food within the group. Four answer categories were allowed: never or seldom, every now and then, regularly, very often.

3- For the food group items 37 and 38 ‘Sugar-sweetened beverages’ and ‘Artificially-sweetened beverages’, participants were asked to score their relative consumption frequency of sugar-sweetened beverages with gas (including diet sodas).

4- For the food group item 9 ‘cooked vegetables’, participants were asked to score their relative consumption frequency of i) soup/broth and ii) cooked vegetables (excluding soup/broth).

5- For the food group item 14 ‘Sausages and other processed meat’, participants were asked to score their relative consumption frequency of i) sausages and ii) processed meat (excluding sausages).

6- For the food group item 25 ‘Butter, sour cream’, participants were asked to score their relative consumption frequency of i) butter and ii) sour cream.

7- For the food group item 26 ‘Margarine, mayonnaise’, participants were asked to score their relative consumption frequency of i) margarine and ii) mayonnaise.

8- For the food group item 31 ‘Sweet snacks, chocolate and pastries’, participants were asked to score their relative consumption frequency of i) sweet cakes and ii) sweet snacks, chocolate and pastries.

9- For the food group item 33 ‘Water’, participants were asked to score their relative consumption frequency of 4 main groups of medium to high levels of bicarbonate mineral water.
