# Supplementary material for: Validity and reproducibility of a short food frequency questionnaire among patients with chronic kidney disease
Source: BMC Nephrol. 2017 Sep 15;18:297. doi: 10.1186/s12882-017-0695-2 (PMC5599889; doi:10.1186/s12882-017-0695-2)

**Additional file 3:** Extract from the questionnaire

1. **French and original version**


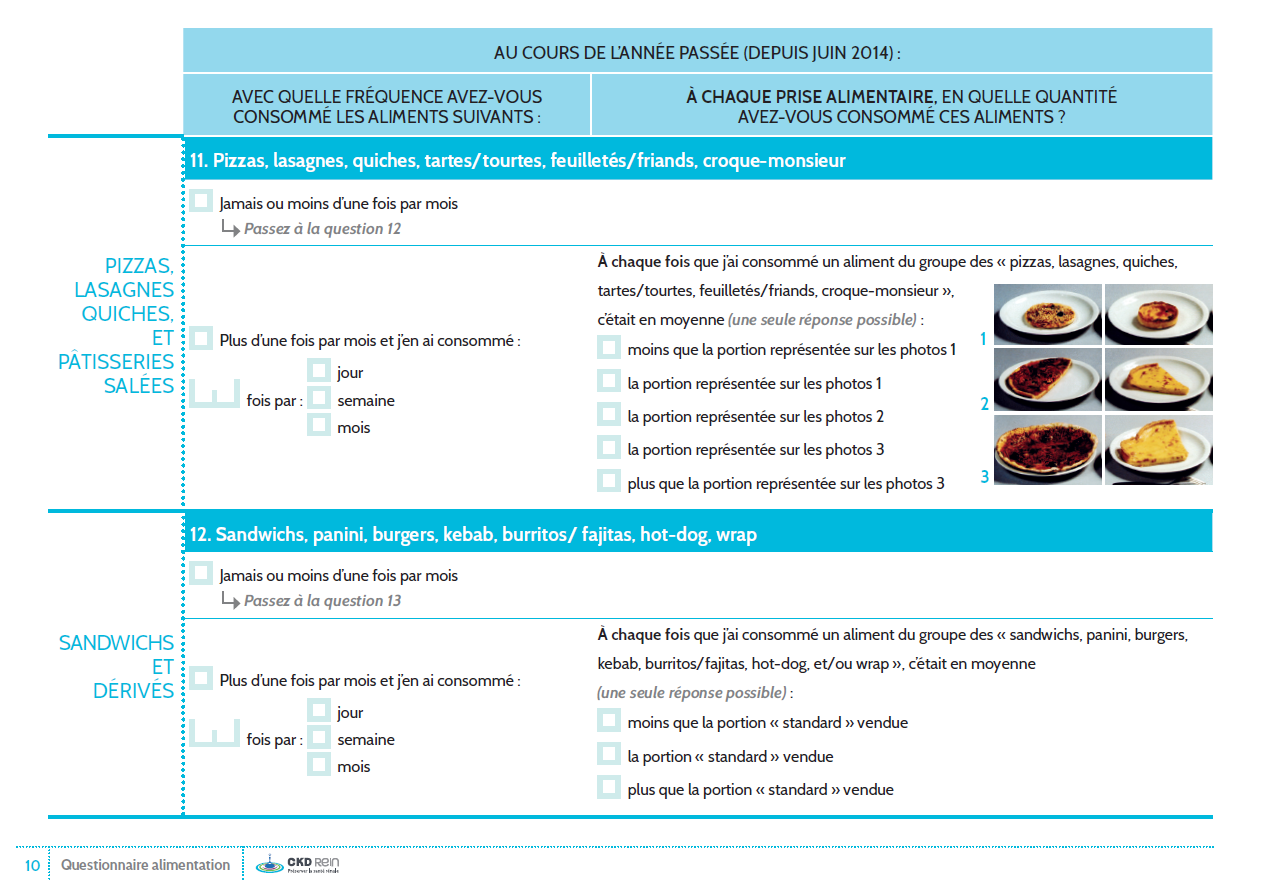


1. **Version translated in English**


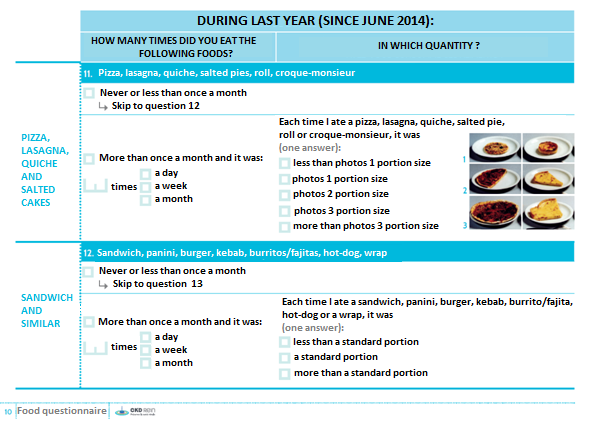

Supplement: Supplementary file 3 — Extract from the questionnaire. (DOCX 331 kb) [file 12882_2017_695_MOESM3_ESM.docx]
