# Supplementary material for: Validity and reproducibility of a short food frequency questionnaire among patients with chronic kidney disease
Source: BMC Nephrol. 2017 Sep 15;18:297. doi: 10.1186/s12882-017-0695-2 (PMC5599889; doi:10.1186/s12882-017-0695-2)
Supplement: Supplementary file 4 — Distribution of EI/BMR according to dietary method and reproducibility/validity study. (DOCX 16 kb) [file 12882_2017_695_MOESM4_ESM.docx]

**Additional file 4:** Distribution of EI/BMR according to dietary method and reproducibility/validity study.

|  |  | **N** | **Min.** | **1st quartile** | **Median** | **3rd quartile** | **Max.** |
| --- | --- | --- | --- | --- | --- | --- | --- |
| **Validity study** | **24hour-recalls** | 127 | 0.51 | 0.94 | 1.12 | 1.32 | 2.36 |
|  | **SFFQ1** | 125 | 0.32 | 0.91 | 1.18 | 1.38 | 2.99 |
|  | **SFFQ2** | 125 | 0.35 | 0.83 | 1.07 | 1.31 | 3.60 |
| **Reproducibility study** | **SFFQ1** | 199 | 0.28 | 0.91 | 1.20 | 1.47 | 4.01 |
|  | **SFFQ2** | 196 | 0.35 | 0.83 | 1.09 | 1.33 | 3.60 |
